# Regulation of the MDM2-p53 pathway by the nucleolar protein CSIG in response to nucleolar stress

**DOI:** 10.1038/srep36171

**Published:** 2016-11-04

**Authors:** Nan Xie, Liwei Ma, Feng Zhu, Wenhui Zhao, Feng Tian, Fuwen Yuan, Jingxuan Fu, Daoyuan Huang, Cuicui Lv, Tanjun Tong

**Affiliations:** 1 Department of Biochemistry and Molecular Biology, School of Basic Medical Sciences, Peking University Health Science Center, Peking University Research Center on Aging, Beijing Key Laboratory of Protein Posttranslational Modifications and Cell Function, 38 Xueyuan Road, Beijing 100191, PR China; 2 Department of Laboratory Animal Science, Peking University Health Science Center, 38 Xueyuan Road, Beijing 100191, PR China

## Abstract

Nucleolar proteins play an important role in the regulation of the MDM2–p53 pathway, which coordinates cellular response to stress. However, the mechanism underlying this regulation remains poorly understood. Here, we report that the nucleolar protein CSIG is a novel and crucial regulator of the MDM2–p53 pathway. We demonstrate that CSIG translocates from the nucleolus to the nucleoplasm in response to nucleolar stress. Moreover, knockdown of CSIG attenuates the induction of p53 and abrogates G1 phase arrest in response to nucleolar stress. CSIG interacts directly with the MDM2 RING finger domain and inhibits MDM2 E3 ubiquitin ligase activity, thus resulting in a decrease in MDM2-mediated p53 ubiquitination and degradation. Our results suggest that the CSIG–MDM2–p53 regulatory pathway plays an important role in the cellular response to nucleolar stress.

The nucleolus is a sub-nuclear compartment that assembles around tandem-repeat ribosomal DNA clusters^1^. Although the nucleolus is primarily associated with ribosome biogenesis, recent evidence has shown that it serves an additional function as a cellular stress sensor in the regulation of the stress response^2^. Ribosome biogenesis comprises three main events: rRNA transcription, rRNA processing, and ribonucleoprotein (RNP) assembly. Disturbing any of the steps during ribosome biogenesis triggers ribosomal stress (also called nucleolar stress)^3^. Nucleolar stress can be caused by different factors, including low-doses of actinomycin D (ActD), which selectively inhibits RNA polymerase I (Pol I), doxorubicin (Dox), which inhibits rRNA transcription, 5-fluorouracil (5-FU), which specifically inhibits late rRNA processing, dysfunctional of nucleolar proteins, and knockdown of the gene encoding Pol I transcription initiation factor-IA (TIF-IA)^4^,^5^.

As one of the primary “gatekeepers” of the cell, p53 plays a major role in sensing and responding to a variety of stresses, thereby maintaining cellular homeostasis^6^. Mouse double minute 2 (MDM2), an E3 ubiquitin ligase, has been identified as a crucial negative regulator of p53 that inhibits p53’s transcriptional activity and promotes its ubiquitination and degradation^7^,^8^,^9^,^10^. MDM2-mediated p53 degradation is essential for the regulation of p53 stability and activation^11^,^12^. Additionally, the E3 ubiquitin ligase activity of MDM2 promotes self-ubiquitination and degradation^13^,^14^.

Emerging evidence has established a critical role of nucleolar proteins in mediating p53 signaling in response to nucleolar stress^15^,^16^. Given the importance of p53 in the response to cellular stress, it is crucial to identify the molecular mechanisms associated with the effects of nucleolar proteins on p53. In response to nucleolar stress, several nucleolar proteins, including RPL5^17^, RPL11^18^,^19^, RPL23^20^,^21^, RPL26^22^, RPL6^23^, RPS7^24^, RPS14^25^, nucleolin (C23)^26^, nucleophosmin (NPM/B23)^27^, alternative reading frame (ARF)^28^, and nucleostemin (NS)^29^, are released into the nucleoplasm, where they bind to MDM2 and inhibit its E3 ubiquitin ligase function, thus leading to p53 stabilization and activation. Previous work has shown that unassembled ribosomal proteins are unstable and are removed from the nucleoplasm by the cellular proteasome system^30^.

Cellular senescence-inhibited gene (CSIG), also known as ribosomal L1 domain containing 1 (RSL1D1), is a nucleolar protein with a ribosomal L1 domain at its N-terminus and a lysine-rich domain at its C-terminus^31^. Previous work has indicated that CSIG is involved in various biological processes, including cellular senescence^32^,^33^, apoptosis^34^, and hepatocellular carcinoma (HCC) proliferation^35^. CSIG also regulates the nucleolar localization of p33ING1 and NS through physical interaction^34^,^36^. However, whether CSIG is involved in the nucleolar stress response remains unknown.

In this study, we found that CSIG translocates into the nucleoplasm in response to nucleolar stress. In addition, CSIG is required for nucleolar stress-induced p53 induction and cell cycle arrest. Our mechanistic analysis revealed that CSIG stabilizes p53 via its interaction with the MDM2 RING finger domain through its ribosomal L1 domain, thus resulting in the inhibition of MDM2-mediated p53 ubiquitination. Our findings indicate that CSIG is a novel and essential regulator of the MDM2-p53 pathway in response to nucleolar stress.

## Results

### CSIG translocates from the nucleolus to the nucleoplasm in response to nucleolar stress

**Figure 1:**
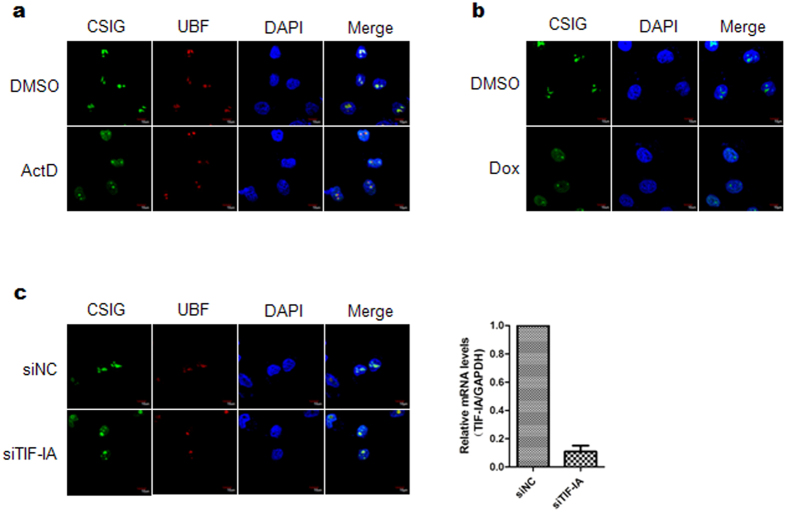
Immunofluorescence staining analysis of endogenous CSIG distribution after ActD, Dox, or TIF-IA siRNA treatment. (**a**) U2OS cells were treated with 5 nM ActD (Sigma) or for 6 h. The cells were fixed and immunostained with anti-CSIG and anti-upstream binding factor (UBF) antibodies. The nuclei were stained with DAPI. Images were obtained by confocal microscopy. UBF was used as a nucleolar marker. (**b**) U2OS cells were treated with 2 μM Dox (Sigma) for 6 h. The cells were fixed and immunostained with anti-CSIG antibody. The nuclei were stained with DAPI. Images were obtained by confocal microscopy. (**c**) U2OS cells were treated with control siRNA (siNC) or TIF-IA siRNA (siTIF-IA) for 72 h. The cells were fixed and immunostained with anti-CSIG and anti-UBF antibodies. The nuclei were stained with DAPI. Images were obtained by confocal microscopy. The right panel shows the knockdown efficiency of siTIF-IA, which was assessed by real-time PCR. Data are presented as the means ± standard deviation (SD).

To determine whether CSIG is involved in the nucleolar stress response, we first examined the localization of CSIG under nucleolar stress triggered by ActD, Dox or knockdown of TIF-IA by using short interfering RNA (siRNA). As shown in Fig. 1, endogenously expressed CSIG localizes predominantly to the nucleolus of control cells; however, CSIG translocates to the nucleoplasm in response to nucleolar stress. Similar results were observed in cells expressing exogenous EGFP-CSIG (Supplementary Fig. S1).

### Nucleolar localization of CSIG depends on rRNA

**Figure 2:**
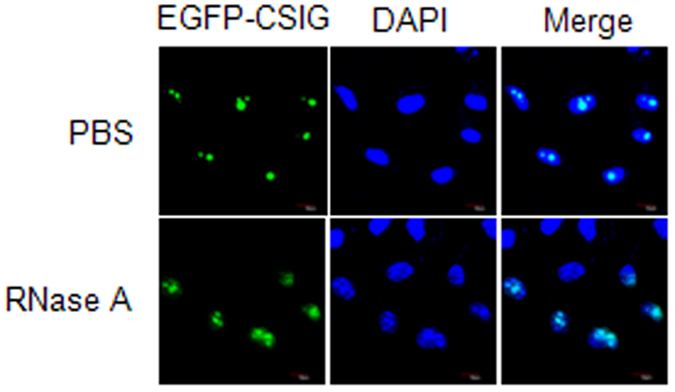
CSIG translocates from the nucleolus to the nucleoplasm in response to RNase A treatment. U2OS cells were transfected with pEGFP-N1-CSIG for 48 h. The RNase A treatment was conducted as previously described^45^. Briefly, permeabilized cells were incubated in the absence or presence of RNase A (1 mg/ml) at 37 °C for 10 min and were subsequently fixed. Nuclei were stained with DAPI. Images were obtained by confocal microscopy.

CSIG is evolutionarily conserved and contains a ribosomal L1 domain at its N-terminus. In bacteria and archaea, L1 functions both as a ribosomal protein that binds rRNA and as a translational repressor that binds its own mRNA^37^. This evidence, together with the observation that suppressing rRNA gene transcription induces the translocation of CSIG from the nucleolus (Fig. 1), suggests that CSIG may be anchored to the nucleolus through rRNA. To evaluate this hypothesis, U2OS cells were permeabilized and incubated with RNase A to digest rRNA. As shown in Fig. 2, RNase A treatment resulted in the translocation of CSIG from the nucleolus to the nucleoplasm. These results suggest that the nucleolar localization of CSIG depends on rRNA.

### CSIG is required for nucleolar stress-induced p53 accumulation and cell cycle arrest

**Figure 3:**
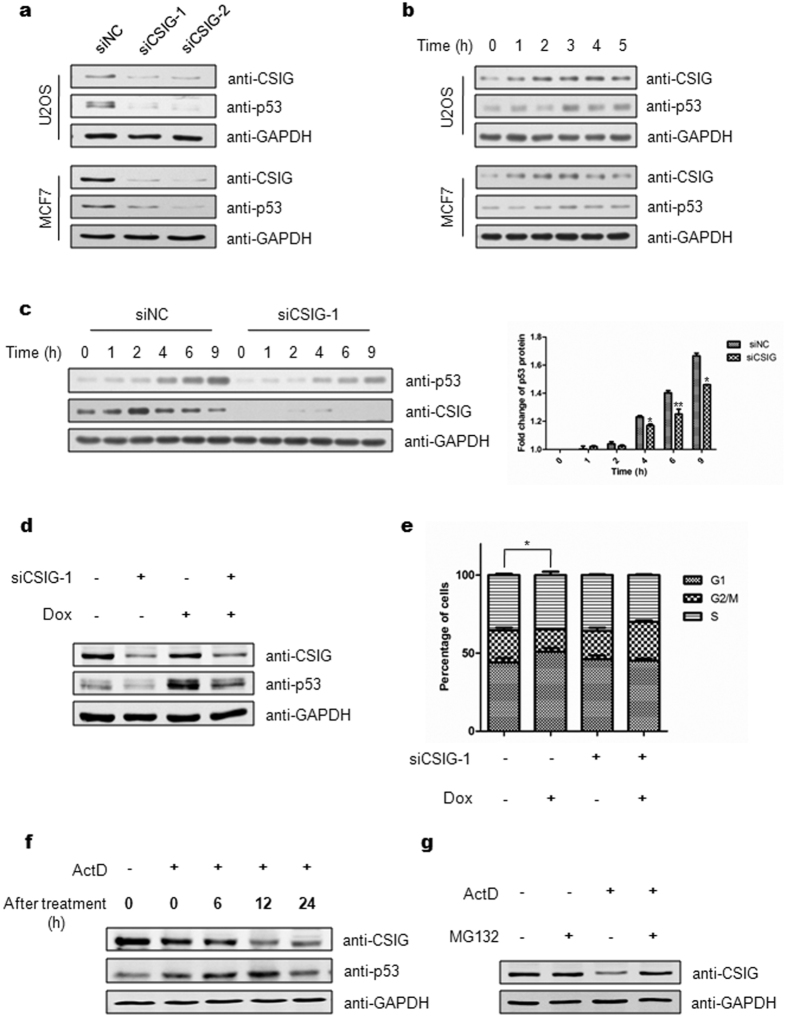
Nucleolar stress causes CSIG-dependent accumulation of p53 and cell cycle arrest. (**a**) U2OS and MCF7 cells were transfected with siCSIG-1, siCSIG-2 or siNC, 72 h after which the cell lysates were subjected to western blot analysis with the indicated antibodies. (**b**) U2OS and MCF7 cells were treated with 5 nM ActD, and the cells were harvested at the indicated time points. Western blot analysis was conducted using the indicated antibodies. (**c**) U2OS cells were transfected with siNC or siCSIG-1 for 72 h, then treated with 5 nM ActD for the indicated time periods. The cell lysates were subjected to western blot analysis using the indicated antibodies. Values represent the fold-change of the p53 protein level. The intensity of p53 band for the untreated cells was set at 1.0 for normalization. Data are presented as the means ± SD. *P < 0.05, **P < 0.01 compared with the siNC group. (**d**) U2OS cells were transfected with siNC or siCSIG-1 for 72 h, then treated with 2 μM Dox for 6 h. The cell lysates were immunoblotted using the indicated antibodies. (**e**) U2OS cells were transfected with siNC or siCSIG-1 for 48 h, then treated with 2 μM Dox for 8 h. Cell cycle analysis was conducted through flow cytometer. Data are presented as the means ± SD. *P < 0.05 compared with G1 phase of the control group. (**f**) U2OS cells were treated with 5 nM ActD for 6 h or were left untreated, after which the culture medium was changed to fresh medium, followed by cluture for the indicated time. The cells were harvested for western blot analysis using the indicated antibodies. (**g**) U2OS cells were treated with 5 nM ActD for 6 h, after which the cells were treated with 20 μM MG132 for an additional 6 h. The cell lysates were subjected to western blot analysis using the indicated antibodies.

Several nucleolar proteins are involved in the stabilization of p53 through their translocation to the nucleoplasm and binding to MDM2. The translocation of CSIG in response to nucleolar stress (Fig. 1) prompted us to explore whether CSIG might also play a role in regulating p53. We first assessed p53 protein levels in U2OS and MCF7 cells, both of which express wild-type p53, after silencing CSIG by using siRNA. As shown in Fig. 3a, depletion of CSIG led to a marked decrease in p53 levels. This result suggests that CSIG is required for maintaining cellular p53 abundance. Next, we analyzed CSIG and p53 expression at different time points in response to ActD treatment in U2OS and MCF7 cells. We found that CSIG was rapidly induced 1 h after treatment, and that p53 levels began to increase 3 h later (Fig. 3b). This correlation suggests a possible relationship between CSIG and p53 in response to ActD treatment. To investigate the role of CSIG in the nucleolar stress-p53 pathway, we analyzed p53 protein expression in cells depleted of CSIG under nucleolar stress. As expected, knockdown of CSIG attenuated ActD-induced p53 accumulation (Fig. 3c). A similar effect of CSIG siRNA on p53 induction in response to Dox treatment was also observed (Fig. 3d). We further tested whether CSIG is crucial for cell cycle arrest in response to nucleolar stress. As shown in Fig. 3e, Dox treatment induced G1 phase arrest in U2OS cells; however, this effect was abrogated after CSIG siRNA transfection.

To gain further insight into the dynamic regulation of CSIG in response to nucleolar stress, U2OS cells were treated with ActD for 6 h, after which the culture medium was changed to fresh medium, followed by cluture for the appropriate time. As shown in Fig. 3f, 12 h after ActD treatment, CSIG levels were significantly lower. Moreover, a significant decrease in p53 was observed 24 h after ActD treatment, which occurred after the decrease in CSIG expression. To test whether the decrease in CSIG and p53 expression occurred as a result of the apoptotic response, U2OS cells were treated as described in Fig. 3f and subjected to apoptosis analysis. Our results showed no significant increase in apoptosis (Supplementary Fig. S2). Therefore, we reasoned that the decrease in CSIG could have resulted from the ActD-induced translocation of CSIG to the nucleoplasm, where CSIG is degraded through a proteasome-dependent pathway. To evaluate this possibility, U2OS cells were treated with 5 nM ActD for 6 h, after which the cells were treated with the proteasome inhibitor MG132 for an additional 6 h. Our results showed that MG132 treatment resulted in significant accumulation of CSIG in the presence of ActD (Fig. 3g). These results suggest that the expression of p53 is decreased after the proteasome-dependent degradation of CSIG in response to nucleolar stress.

### CSIG inhibits the proteasomal degradation of p53

**Figure 4:**
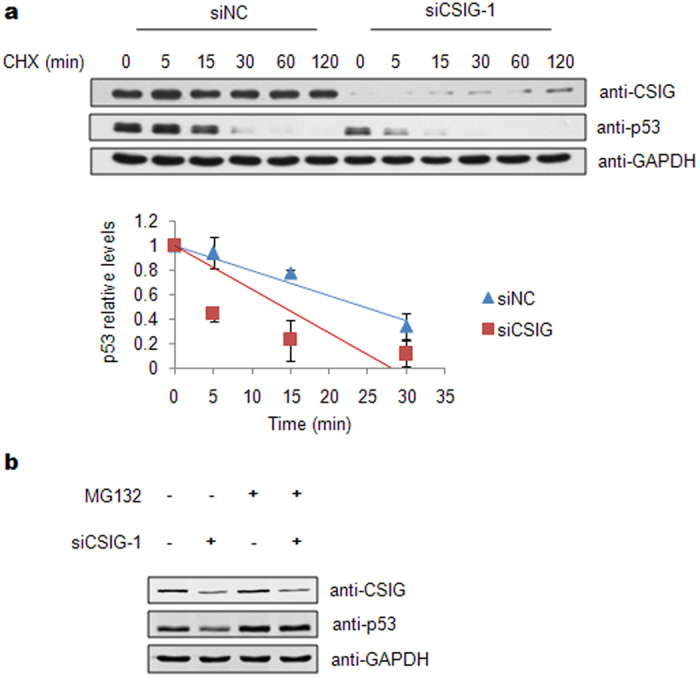
CSIG depletion accelerates the proteasomal degradation of p53. (**a**) U2OS cells were transfected with siRNAs as indicated. 72 h after transfection, the cells were treated with 100 μg/ml CHX (Sigma) for the indicated periods of time. Western blot analysis was performed, and the intensity of p53 band for the untreated cells was set at 1.0 for normalization. Data are presented as the means ± SD. (**b**) U2OS cells were transfected with siRNAs as indicated. 72 h after transfection, cells were treated with 20 μM MG132 or DMSO for 6 h. Then the cells were harvested and subjected to western blot analysis with the indicated antibodies.

To elucidate the mechanism underlying CSIG regulation of p53, we tested whether CSIG affects p53 protein stability. U2OS cells transfected with either control or CSIG siRNA were treated with the protein synthesis inhibitor cycloheximide (CHX), and the p53 protein levels were examined at the indicated time points. As shown in Fig. 4a, the p53 half-life was ~25 min in cells transfected with control siRNA. Knockdown of CSIG decreased the half-life of p53 to ~15 min. We also found that the effect of CSIG siRNA on p53 protein stability was abrogated when the cells were treated with MG132 (Fig. 4b). These results demonstrate that CSIG depletion accelerates the proteasomal degradation of p53.

### CSIG inhibits MDM2-mediated p53 degradation

**Figure 5:**
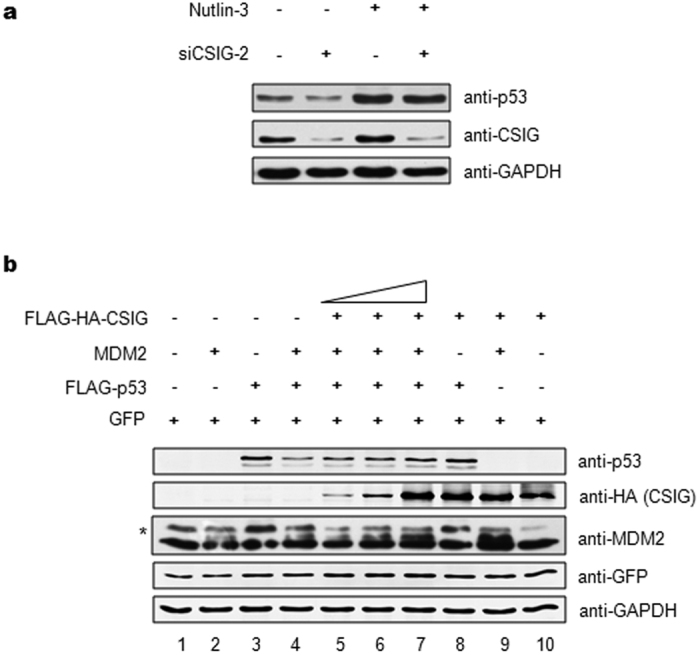
CSIG inhibits MDM2-mediated p53 degradation. (**a**) MCF7 cells were transfected with siCSIG or siNC as indicated. At 48 h after transfection, cells were treated with 5 μM nutlin-3 (Selleck) for 12 h. Then the cells were harvested and subjected to western blot analysis with the indicated antibodies. (**b**) H1299 cells were co-transfected with pIRES-FLAG-p53, pCMV-MDM2, and increasing amounts of pIRES-FLAG-HA-CSIG as indicated. pEGFP-N1 was used as a transfection efficiency control. Western blot analysis was conducted at 24 h post-transfection. The asterisk indicates the specific MDM2 band.

The observation that CSIG regulates the proteasomal degradation of p53 encouraged us to determine whether CSIG regulates p53 through MDM2. Using the MDM2 inhibitor nutlin-3, we found that p53 levels were unchanged in cells transfected with CSIG siRNA relative to those transfected with control siRNA (Fig. 5a). To further test whether CSIG stabilizes p53 by inhibiting MDM2-mediated p53 degradation, H1299 cells, which lack endogenous p53, were co-transfected with pIRES-FLAG-p53, pCMV-MDM2, and increasing doses of pIRES-FLAG-HA-CSIG. As shown in Fig. 5b, MDM2 transfection resulted in the degradation of p53, as expected (lane 4); however, this effect was attenuated by CSIG transfection in a dose-dependent manner (lanes 5–7). Moreover, we observed that the expression of CSIG also resulted in the stabilization of MDM2 (compare lane 9 with lane 2).

### CSIG stabilizes p53 by inhibiting MDM2-mediated p53 ubiquitination *in vivo* and *in vitro*

**Figure 6:**
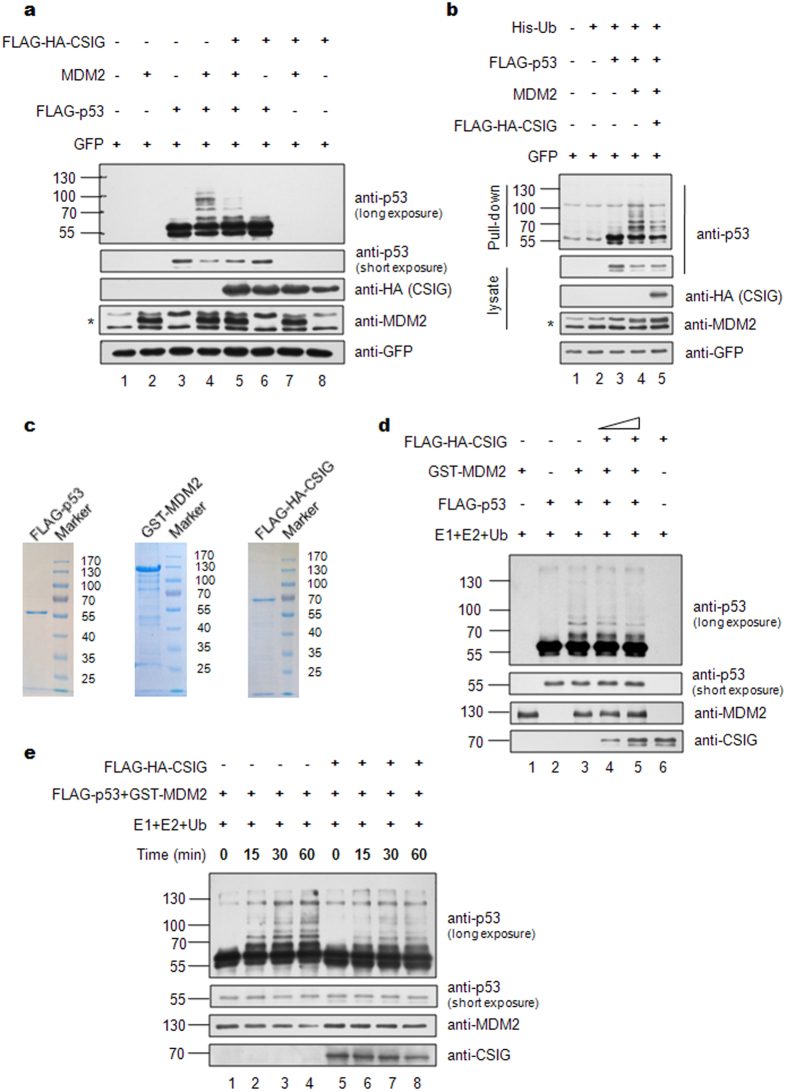
CSIG inhibits MDM2-mediated ubiquitination of p53. (**a**) H1299 cells were transfected with the indicated plasmids. 24 h after transfection, the cells were treated with 20 μM MG132 for 6 h. Then, the cells were lysed and subjected to western blot analysis with the indicated antibodies. The asterisk indicates the specific MDM2 band. (**b**) H1299 cells were transfected with the indicated plasmids for 24 h. The cells were incubated with 20 μM MG132 for 6 h before harvesting, after which the cells were lysed under denaturing conditions and incubated with Ni-NTA Agarose (QIAGEN). Cell lysates and Ni-NTA Agarose-bound proteins were analyzed by western blotting with the indicated antibodies. The asterisk indicates the specific MDM2 band. (**c**) SDS-PAGE analysis and Coomassie blue staining of the purified components used for *in vitro* ubiquitination experiments. (**d**) For *in vitro* ubiquitination experiments, purified FLAG-p53 was incubated at 37 °C for 1 h with E1, E2, Ub, GST-MDM2, and increasing amounts of purified FLAG-HA-CSIG as indicated, and then analyzed by western blotting. (**e**) Purified FLAG-p53 was mixed with E1, E2, Ub and GST-MDM2 in the absence (lanes 1–4) or presence (lanes 5–8) of FLAG-HA-CSIG for *in vitro* ubiquitination reactions. The mixture was incubated at 37 °C for the indicated amount of time, and then the reactions were analyzed by western blotting.

To explore whether CSIG is involved in MDM2-mediated ubiquitination of p53, we conducted ubiquitination assays. As shown in Fig. 6a,b, although MDM2 transfection resulted in an increase in ubiquitinated p53 (lane 4), ubiquitinated p53 levels were markedly decreased in the presence of CSIG, and the steady-state level of p53 was correspondingly increased (lane 5). To further confirm this observation, *in vitro* ubiquitination assays were performed by using purified proteins (Fig. 6c). As shown in Fig. 6d,e, MDM2 promoted the ubiquitination of p53; however, in the presence of CSIG, MDM2 did not ubiquitinate p53 to the same extent. These results demonstrate that CSIG directly inhibits MDM2-mediated p53 ubiquitination. Consistently with the results in Fig. 5b, CSIG also inhibited MDM2 degradation (Fig. 6e).

### CSIG interacts with MDM2

**Figure 7:**
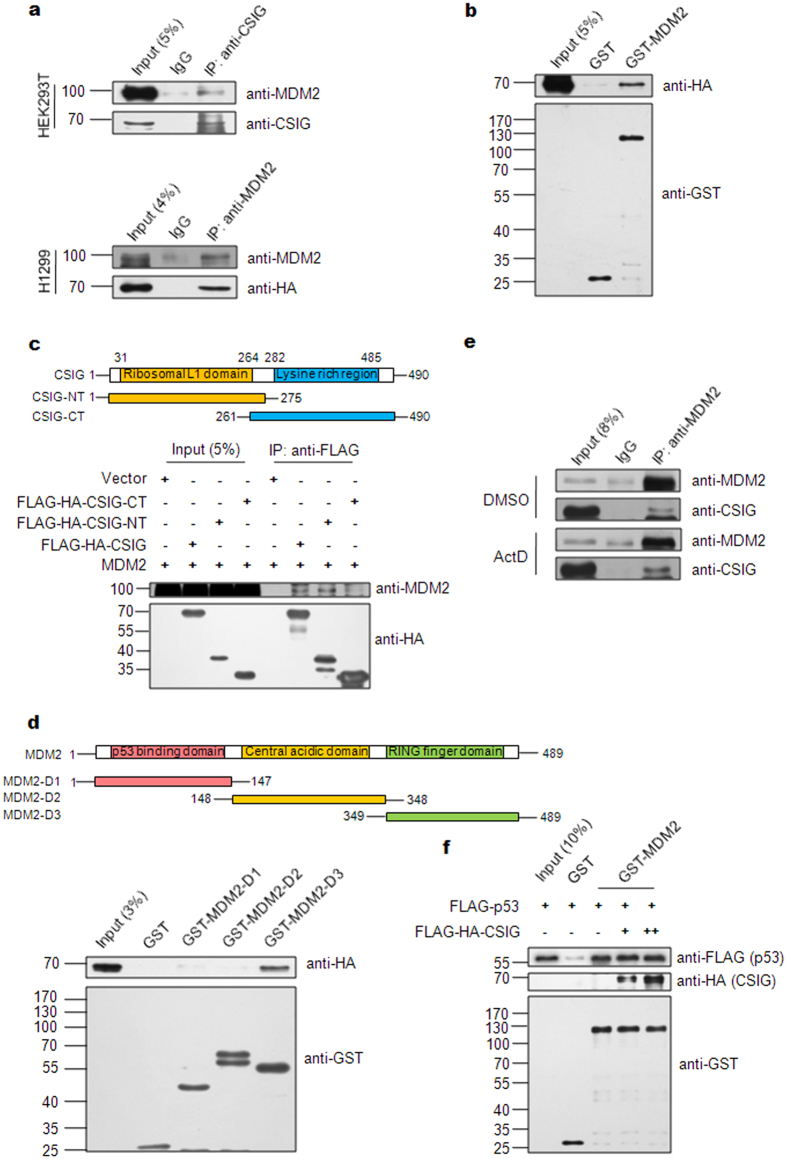
CSIG interacts with MDM2. (**a**) HEK293T or H1299 cells were co-transfected with pCMV-MDM2 and pIRES-FLAG-HA-CSIG for 48 h. The cell lysates were subjected to IP using the indicated antibodies and then analyzed by western blotting. (**b**) Purified GST or GST-MDM2 proteins were incubated separately with FLAG-HA-CSIG. The bound proteins were separated using Glutathione Sepharose and were detected by western blotting. (**c**) Schematic representation of the full-length CSIG protein (amino acids 1–490) and CSIG truncation mutants (CSIG-NT: amino acids 1–275, which includes the ribosomal L1 domain; CSIG-CT: amino acids 261–490, which includes the lysine-rich domain). HEK293T cells were co-transfected with pCMV-MDM2 and either pIRES-FLAG-HA-CSIG, pIRES-FLAG-HA-CSIG-NT, or pIRES-FLAG-HA-CSIG-CT for 48 h, after which the cell lysates were subjected to IP with the indicated antibodies and then analyzed by western blotting. (**d**) Schematic representation of the full-length MDM2 protein (amino acids 1–489) and MDM2 truncation mutants (MDM2-D1: amino acids 1–147, which includes the p53 binding domain; MDM2-D2: amino acids 148–348, which includes the central acidic domain; MDM2-D3: amino acids 349–489, which includes the RING finger domain). Purified GST, GST-MDM2-D1, GST-MDM2-D2, or GST-MDM2-D3 proteins were incubated separately with FLAG-HA-CSIG. The bound proteins were separated using Glutathione Sepharose and were analyzed by western blotting. (**e**) U2OS cells were treated with 5 nM ActD for 6 h, then with 20 μM MG132 for an additional 6 h. The cells were then harvested and subjected to IP using the indicated antibodies and then analyzed by western blotting. (**f**) Purified GST or GST-MDM2 proteins were incubated separately with FLAG-p53 and an increasing amount of FLAG-HA-CSIG as indicated. The bound proteins were separated using Glutathione Sepharose and were analyzed by western blotting.

To explore the mechanism underlying CSIG regulation of the MDM2-p53 pathway, we next examined whether CSIG interacts with MDM2. Immunoprecipitation (IP) of whole cell extracts from HEK293T cells transfected with pCMV-MDM2 and pIRES-FLAG-HA-CSIG showed that MDM2 co-immunoprecipitated with CSIG (Fig. 7a), thus suggesting that these proteins associate with each other in a cellular context. To test whether the interaction between CSIG and MDM2 is mediated by p53, we performed an IP assay in p53-null H1299 cells. Our results showed that CSIG co-immunoprecipitated with MDM2 (Fig. 7a), thus suggesting that the interaction between CSIG and MDM2 is p53-independent. We also observed direct binding between CSIG and MDM2 via GST pull-down assays (Fig. 7b). Next, we generated and analyzed truncation mutants to identify the domain of CSIG responsible for MDM2 binding. As shown in Fig. 7c, MDM2 interacted most strongly with the CSIG-NT construct, which contains the ribosomal L1 domain. Furthermore, we tested MDM2 truncation constructs to determine the domain of MDM2 required for CSIG binding. We found that the MDM2-D3 construct, which contained the RING finger domain (responsible for E3 ligase activity), was critical for MDM2 interaction with CSIG (Fig. 7d). Importantly, we observed that the interaction between CSIG and MDM2 was enhanced after ActD treatment (Fig. 7e). Given that CSIG inhibits MDM2-mediated p53 degradation (Fig. 5) and ubiquitination (Fig. 6), we wondered whether the interaction between CSIG and MDM2 might interrupt the MDM2-p53 interaction. As shown in the results of our GST pull-down assay in Fig. 7f, CSIG had no effect on the MDM2-p53 interaction.

## Discussion

Recent studies have begun to unveil the extra-ribosomal functions of nucleolar proteins. The critical role of nucleolar proteins in the regulation of p53 in response to nucleolar stress has attracted considerable attention. Given the importance of p53 in the response to cellular stress, it is crucial to identify the molecular mechanisms underlying the roles of nucleolar proteins in the regulation of p53. Several nucleolar proteins, including RPL5^17^, RPL11^18^,^19^, RPL23^20^,^21^, RPL26^22^, RPL6^23^, RPS7^24^, RPS14^25^, C23^26^, NPM/B23^27^, ARF^28^, and NS^29^, have been reported to stabilize p53 by binding to MDM2 and blocking MDM2-mediated p53 ubiquitination and degradation in response to nucleolar stress.

Our previous work has indicated that CSIG, a nucleolar protein, exerts important extra-ribosomal functions, including the regulation of cellular senescence^32^,^33^, apoptosis^34^, and HCC proliferation^35^. CSIG also regulates the nucleolar localization of p33ING1 and NS through physical interaction^34^,^36^.

**Figure 8:**
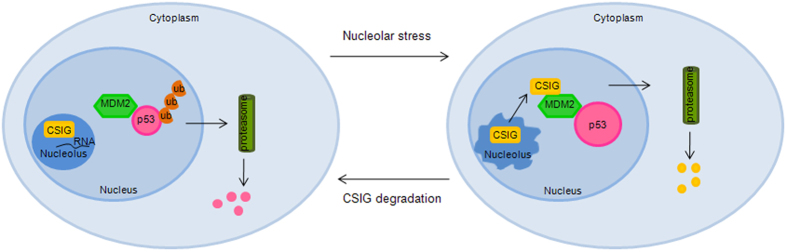
Schematic diagram demonstrating the role of CSIG in the regulation of the MDM2-p53 pathway. Under normal conditions, p53 is strictly maintained at low levels by MDM2, and CSIG predominantly localizes to the nucleolus in a RNA-dependent manner (left panel). In response to nucleolar stress, CSIG translocates into the nucleoplasm, where it interacts with MDM2 and inhibits MDM2-mediated p53 ubiquitination and degradation (right panel). The nucleoplasmic CSIG is degraded gradually. Decreased levels of CSIG allow p53 to return to basal levels.

In this study, we identified CSIG is a novel and crucial regulator of p53 through the modulation of MDM2 E3 ubiquitin ligase activity. As summarized in Fig. 8, CSIG translocates from the nucleolus into the nucleoplasm in response to nucleolar stress, where it binds the MDM2 RING finger domain through its ribosomal L1 domain. This interaction blocks MDM2-mediated p53 ubiquitination and degradation, and CSIG is degraded gradually in the nucleoplasm. The decrease in CSIG levels in turn relieves its inhibitory effect on MDM2 and allows p53 to return to basal levels.

Several nucleolar proteins stabilize p53 by binding to and blocking MDM2 E3 ligase activity toward p53. Most of the reported nucleolar proteins bind to the central acid domain of MDM2; however, our study shows that CSIG binds to the RING finger domain of MDM2, which is responsible for MDM2 E3 ubiquitin ligase activity (Fig. 7d). CSIG-MDM2 binding is similar to the binding patterns observed between MDM2 and RPL23, RPS15 and RPS20^38^, thus suggesting that nucleolar proteins use distinct mechanisms in the regulation of p53. Moreover, we observed that CSIG also inhibits MDM2 degradation (Figs 5b and 6e). Because MDM2 can ubiquitinate itself^13^,^14^, we speculate that the ability of CSIG to inhibit MDM2-dependent p53 ubiquitination is most probably related to its ability to inhibit the E3 ubiquitin ligase activity of MDM2. Additionally, as with RPL11^39^, CSIG binding to MDM2 may induce a conformational change in MDM2, thus making the interaction between p53 and the RING domain of MDM2 more difficult and thereby impairing the ubiquitination of p53 by MDM2. To evaluate this hypothesis, a 3D structure of the CSIG-MDM2-p53 ternary complex is needed.

Although we demonstrated that CSIG alone was sufficient to interact with MDM2 (Fig. 7b) and inhibit MDM2-mediated p53 ubiquitination (Fig. 6d,e), further research will be needed to illustrate the interrelationship between CSIG and other nucleolar proteins during this process. It is possible that CSIG may recruit and facilitate other regulators, such as RPL5, RPL11, RPL23, or NS, which bind to MDM2. Another possibility is that the CSIG-MDM2-p53 pathway and other nucleolar protein-MDM2-p53 pathways are non-redundant and possess similar abilities to stabilize p53.

Takagi, M. *et al*. have reported that RPL26 regulates p53 translation by binding the 5′-untranslated region (UTR) of p53 mRNA^40^. We have observed that CSIG regulates PTEN by interacting with the 5′-UTR of PTEN mRNA^32^. Whether the regulation of p53 by CSIG also occurs at the post-transcriptional level is worthy of further study. RPL11 and RPS14 are regulated by NEDD8^41^,^42^. The NEDDylation of RPS14 and RPL11 influences their subcellular localization and stability in response to nucleolar stress. Nevertheless, it remains unknown whether NEDDylation can also occur on CSIG, which is an interesting possibility, given that we observed that nucleolar stress triggers proteasomal degradation of CSIG (Fig. 3g).

Velimezi, G. *et al*. have shown that ataxia telangiectasia mutated (ATM), a key DNA-damage-response (DDR) kinase and tumor suppressor, negatively regulates ARF in response to DDR^43^. It brings up a more complex function of nucleolar protein in response to nucleolar and genotoxic stress. There is an interest in studying the relationship between CSIG and DDR. It would be important to further investigate whether CSIG is needed for p53 stability under genotoxic stimuli, or CSIG’s regulatory effect on p53 is specific to the nucleolar stress-CSIG-MDM2-p53 pathway.

In summary, in this study, we identified a novel nucleolar regulator of the MDM2–p53 pathway. We show that CSIG translocates from the nucleolus and stabilizes p53 by binding to the MDM2 RING finger domain and inhibiting its E3 ubiquitin ligase activity in response to nucleolar stress. The results of our study provide a better understanding of the molecular mechanisms underlying the role of nucleolar proteins in the regulation of p53.

## Methods

### Cell culture & transfections

U2OS, MCF7, H1299, and HEK293T cells were maintained in DMEM (Macgene) supplemented with 10% FBS, 100 U/ml penicillin, and 100 μg/ml streptomycin at 37 °C in a humidified atmosphere under 5% CO_2_.

Plasmid transfections were performed with Lipofectamine 2000 (Invitrogen) according to the manufacturer’s instructions. The following plasmids were used in this study: pEGFP-N1, pEGFP-N1-CSIG, pIRES-FLAG-HA-CSIG, pIRES-FLAG-HA-CSIG-NT, and pIRES-FLAG-HA-CSIG-CT, which were described previously^32^; pCMV-MDM2, pIRES-FLAG-p53, pET-FLAG-p53, and pcDNA3.1-His-Ub, which were kindly provided by Dr Zhao; and GST-MDM2, GST-MDM2-D1, GST-MDM2-D2, and GST-MDM2-D3, which were constructed by cloning full-length or truncated MDM2 from pCMV-MDM2 into the pGEX-4T-2 vector.

siRNA transfections were performed using Lipofectamine RNAi MAX Reagent (Invitrogen). Cells were transfected with siRNA twice during 24 h intervals. The sequences of the siRNAs were as follows: CSIG-1 siRNA, 5′-AGAAGGAACAGACGCCAGA-3′; CSIG-2 siRNA, 5′-UUAUCCAGCUGCUUCCGUGCUGUCG-3′; TIF-IA siRNA, 5′-CGACACCGUGGUUUCUCAUGCCAAU-3′; and control siRNA, 5′-UUCUCCGAACGUGUCACGU-3′.

### Western blotting & antibodies

Cells were lysed in RIPA buffer (Macgene) supplemented with a protease inhibitor cocktail (Amresco). Protein concentrations were evaluated using BCA Protein Assay Kit (Thermo Scientific). Western blotting was performed as previously described^32^.

The following antibodies were used in this study: Anti-GAPDH (1A6, Bioworld), Anti-p53 (DO-1, Santa Cruz), Anti-GFP (B-2, Santa Cruz), Anti-MDM2 (2A10, Abcam), Anti-HA (C29F4, CST), Anti-FLAG (M2, Sigma), Anti-GST (3B2, MBL), Anti-CSIG (previously described^32^), and Anti-UBF (F-9, Santa Cruz).

### Immunoprecipitation (IP)

Cells were harvested and lysed in FLAG lysis buffer (50 mM Tris-HCl pH7.3, 137 mM NaCl, 1% Triton X-100, 10% glycerol, 1 mM NaF, 1 mM Na_3_VO_4_, 1 mM DTT, 1 mM PMSF, and protease inhibitor cocktail). The supernatant was incubated with the appropriate antibody overnight at 4 °C, and then Protein G Sepharose (GE Healthcare) were added to the samples and incubated for another 4 h at 4 °C. The immunoprecipitates were washed three times with FLAG lysis buffer and then boiled. The samples were resolved via SDS-PAGE and were probed using the indicated antibodies.

### Immunofluorescence

Cells grown on coverslips were fixed in 4% paraformaldehyde for 10 min, washed with PBS, and permeabilized with 0.5% Triton X-100 for 10 min at room temperature. Samples were blocked with 10% goat serum for 1 h at room temperature and then incubated with the indicated antibodies overnight at 4 °C. The samples were washed with PBS and incubated with Alexa Fluor^®^ 647- or Alexa Fluor^®^ 488-conjugated secondary antibody (Abcam) for 1 h at room temperature. The samples were washed again with PBS and were stained with DAPI (Sigma). Immunofluorescence images were captured with a confocal laser scanning microscope (Olympus FV1000).

### Real-time PCR

Total RNA was isolated from cells with RNeasy Mini Kit (QIAGEN) according to the manufacturer’s instructions. First-strand cDNA was synthesized using a TransScript First-Strand cDNA Synthesis kit (Transgen). Real-time PCR analysis was performed using SYBR Select Master Mix (Life technologies) in conjunction with an ABI Prism 7500 Sequence Detection System. The data were analyzed using the ΔΔCT method. Primer sequences used in this assay were as follows: TIF-IA, forward 5′-CATCCCGGCAGGGTATTGAA-3′, reverse 5′-CCTTCCGTGGAATCTGTCCC-3′; GAPDH, forward 5′-CGACCACTTTGTCAAGCTCA-3′, reverse 5′-AGGGGTCTACATGGCAACTG-3′.

### Cell cycle analysis

Cells were harvested with trypsin and fixed in 70% ethanol overnight at 4 °C, after which the cells were treated with RNase A (100 μg/ml, Sigma) at 37 °C for 30 min and stained with propidium iodide (20 μg/ml, Millipore). The cells were then analyzed for DNA content using a BD FACS flow cytometer. Data were analyzed using the Cell Quest and Modfit software programs.

### Protein purification & GST pull-down

To purify FLAG-HA-CSIG protein, the corresponding plasmid was transfected into H1299 cells, 48 h after which the cells were lysed by sonication in BC500 buffer (25 mM Tris-HCl pH7.3, 500 mM NaCl, 0.5% Triton X-100 and 20% glycerol). The soluble extracts were immunoprecipitated with ANTI-FLAG M2 Affinity Gel (Sigma). The beads were washed with BC500 buffer once, and further washed with BC100 buffer (25 mM Tris-HCl pH7.3, 100 mM NaCl, 0.5% Triton X-100 and 20% glycerol) three times. Proteins were then eluted with FLAG Peptide (Sigma).

GST, GST-MDM2, GST-MDM2-D1, GST-MDM2-D2, GST-MDM2-D3, and FLAG-p53 were expressed in Rosetta (DE3) *E*. *coli* cells induced with IPTG (1 mM). Cell pellets were resuspended in BC500 buffer containing DTT (1 mM) and then were disrupted by sonication. Cleared cell lysates were incubated with Glutathione Sepharose (GE Healthcare, for GST, GST-MDM2, GST-MDM2-D1, GST-MDM2-D2, and GST-MDM2-D3) or ANTI-FLAG M2 Affinity Gel (for FLAG-p53). After extensive washing, the bound protein was eluted with Reduced Glutathione (Amresco) or FLAG Peptide.

To perform GST pull-down analysis, purified GST, GST-MDM2, GST-MDM2-D1, GST-MDM2-D2, or GST-MDM2-D3 proteins were incubated with purified FLAG-HA-CSIG and/or FLAG-p53 protein overnight at 4 °C. Glutathione Sepharose were added and incubated for 5 h. The beads were washed with BC100 buffer and boiled. The samples were resolved by SDS-PAGE and analyzed by western blotting using the indicated antibodies.

### *In vivo* ubiquitination assay

H1299 cells were transfected with pIRES-FLAG-p53, pCMV-MDM2, and pIRES-FLAG-HA-CSIG in the presence or absence of pcDNA3.1-His-Ub. 24 h after transfection, the cells were treated with MG132 (20 μM, Selleck) for 6 h. In the absence of His-Ub, the cell lysates were subjected to western blot analysis with the indicated antibodies. When His-Ub was used, the cells were harvested and split into two aliquots, one for western blot analysis and the other for Ni-NTA pull-down assay. The Ni-NTA pull-down assay was conducted as previously described^44^.

### *In vitro* ubiquitination assay

Purified FLAG-p53, GST-MDM2, and FLAG-HA-CSIG proteins were mixed with 12 ng UBE1 (E1, Boston Biochem), 200 ng UbcH5c (E2, Boston Biochem), and 2 μg Ubiquitin (Ub, Boston Biochem) in 20 μl of 1× Energy Regeneration Solution (ERS, Boston Biochem). The mixture was incubated at 37 °C for indicated time. The reactions were stopped by addition of SDS loading buffer, after which the reactions were analyzed by western blotting.

### Statistical analysis

Data are presented as the means ± SD. The Student’s t-test was used to analyze statistical differences between groups. A two-tailed P-value of less than 0.05 was considered significant.

## Additional Information

**How to cite this article**: Xie, N. *et al*. Regulation of the MDM2-p53 pathway by the nucleolar protein CSIG in response to nucleolar stress. *Sci. Rep.* **6**, 36171; doi: 10.1038/srep36171 (2016).

**Publisher’s note:** Springer Nature remains neutral with regard to jurisdictional claims in published maps and institutional affiliations.

## Supplementary Material

Supplementary Information
