# Supplementary material for: Regulation of the MDM2-p53 pathway by the nucleolar protein CSIG in response to nucleolar stress
Source: Sci Rep. 2016 Nov 4;6:36171. doi: 10.1038/srep36171 (PMC5095888; doi:10.1038/srep36171)
Supplement: Supplementary Information [file srep36171-s1.pdf]

Supplementary Information

Regulation of the MDM2-p53 pathway by the nucleolar protein CSIG in response to nucleolar stress

Nan Xie[1,+], Liwei Ma[1,+], Feng Zhu[1], Wenhui Zhao[1], Feng Tian[2], Fuwen Yuan[1], Jingxuan Fu[1], Daoyuan Huang[1], Cuicui Lv[1], and Tanjun Tong[1,*]

[1]Department of Biochemistry and Molecular Biology, School of Basic Medical Sciences, Peking University Health Science Center, Peking University Research Center on Aging, Beijing Key Laboratory of Protein Posttranslational Modifications and Cell Function, 38 Xueyuan Road, Beijing 100191, PR China.

[2]Department of Laboratory Animal Science, Peking University Health Science Center, 38 Xueyuan Road, Beijing 100191, PR China.

[+] These authors contributed equally to this work.

[*]Correspondence and requests for materials should be addressed to T. T. (Email: ttj@bjmu.edu.cn)

Apoptosis analysis

Cells were harvested with trypsin and washed twice in PBS. Cells were then resuspended in cold binding buffer, after which the cells were stained with Annexin V-FITC and propidium iodide at room temperature for 15 min. The cells were then analyzed using a flow cytometry.

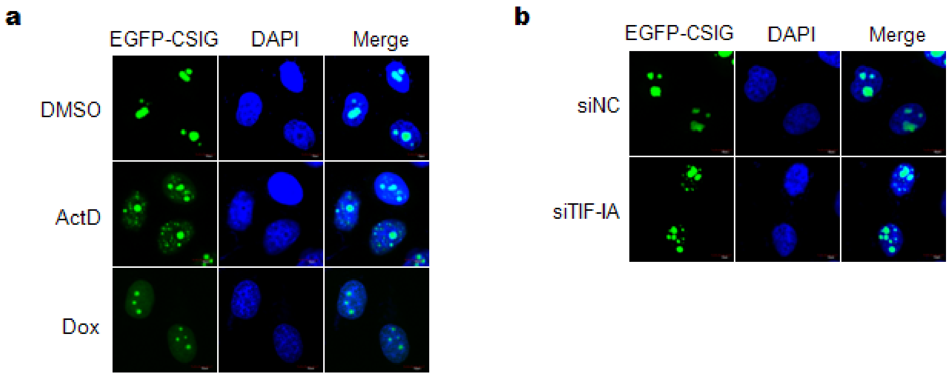

Supplementary Figure S1. Immunofluorescence staining analysis of exogenous EGFP-CSIG distribution after ActD, Dox, or TIF-IA siRNA treatment. a. U2OS cells were transfected with pEGFP-N1-CSIG for 48h, then treated with 5 nM ActD or 2 μM Dox for 6 h. The cells were fixed and nuclei were stained with DAPI. Images were obtained by confocal microscopy. **b.** U2OS cells were transfected with pEGFP-N1-CSIG. The cells were treated with siNC or siTIF-IA for 72 h and were fixed. Nuclei were stained with DAPI. Images were obtained by confocal microscopy.

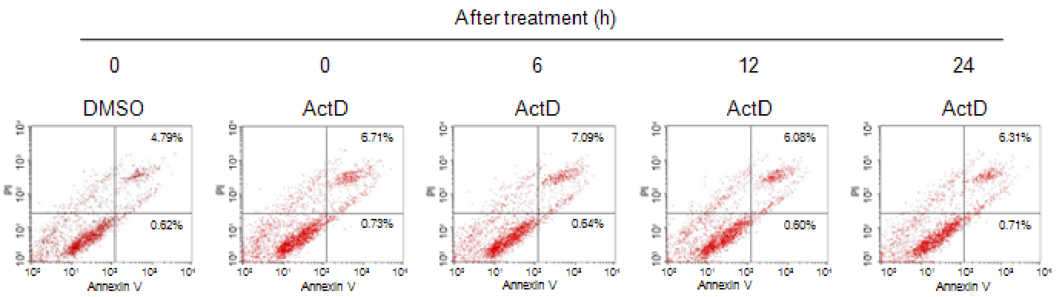

Supplementary Figure S2. Analysis of cell apoptosis after ActD treatment. U2OS cells were treated as in Figure 3f. Apoptotic cells were anayzed by apoptosis analysis.
